# Clinical Characteristics of Fever After Extracorporeal Membrane Oxygenation Decannulation: Differentiating Infectious from Non-Infectious Causes of Fever and Their Impact on Outcomes

**DOI:** 10.3390/jcm14010059

**Published:** 2024-12-26

**Authors:** Sua Kim, Jooyun Kim, Saeyeon Kim, Ji-Hee Lee, YuJin Kim, Jinwook Hwang, Jae Seung Shin, Je Hyeong Kim

**Affiliations:** 1 Department of Critical Care Medicine, Korea University Ansan Hospital, Korea University College of Medicine, Ansan 15355, Republic of Korea; sua0047@gmail.com (S.K.); jooyuna777@gmail.com (J.K.); cecillia2@gmail.com (S.K.); cloud9ljh@naver.com (J.-H.L.); 2 Division of Pulmonology, Department of Internal Medicine, Korea University Ansan Hospital, Korea University College of Medicine, Ansan 15355, Republic of Korea; gene2001@korea.ac.kr; 3 Department of Thoracic and Cardiovascular Surgery, Korea University Ansan Hospital, Korea University College of Medicine, Ansan 15355, Republic of Korea; znuke75@korea.ac.kr (J.H.); jason@korea.ac.kr (J.S.S.)

**Keywords:** fever, systemic inflammatory response syndrome, extracorporeal membrane oxygenation, decannulation, infection

## Abstract

**Background:** A fever is an important sign that affects patient outcomes with various etiologies in the post-decannulation period of extracorporeal membrane oxygenation (ECMO); however, the cause is not fully understood. This study aimed to investigate the characteristics and clinical implications of fevers after ECMO decannulation in critically ill patients. **Methods:** We conducted a retrospective, single-center study of adult patients who were successfully weaned off venoarterial (VA) or venovenous (VV) ECMO. Decannulation fever was defined as fever that occurred within 72 h of ECMO decannulation. The peak and duration of fever were followed for 2 weeks after decannulation, and the relationship with infection was assessed. **Results:** A total of 47 patients were included (22 [46.8%] on VA ECMO and 25 [53.2%] on VV ECMO). There were 35 (74.5%) patients who had decannulation fever, including 16 (34%) with active infections. Active infection during the study period was not related to the ECMO setting or duration; rather, infectious fever lasted longer than non-infectious fever (4 [interquartile range; IQR: 1–7] vs. 11 [IQR: 2–7] days, *p* = 0.023), and the C-reactive protein level was higher on post-decannulation day 7 (*p* = 0.006). Active infection was associated with increased mortality (odds ratio [OR] 6.067, 95% confidence interval [CI] 1.1289–32.644, *p* = 0.036), whereas decannulation fever was not (OR 0.156, 95% CI 0.025–0.977, *p* = 0.047). **Conclusions:** Fever is an important indicator of ECMO decannulation. However, the different timing and duration of fevers during the post-decannulation period of ECMO may have various clinical implications.

## 1. Introduction

Fever is one of the most common signs observed during hospitalization and can indicate various conditions in patients [1]. In intensive care units (ICUs), fever is often a sign of an infectious condition that can lead to severe or even fatal outcomes if not promptly identified and treated [2,3]. Consequently, physicians are particularly vigilant when a patient recovering from the most critical condition develops a fever. The evaluation of fever in ICU patients typically begins with diagnostic investigations of the infectious condition and the initiation of empirical antibiotic therapy [1,4].

Extracorporeal membrane oxygenation (ECMO) can trigger systemic inflammatory response syndrome (SIRS) regardless of infectious causes, as exposure of blood to the ECMO circuit activates both the immune and coagulation systems [5,6]. Therefore, physicians encounter a considerable number of patients who develop fever after ECMO decannulation [7,8], and the complex clinical background of these patients often hinders the differential diagnosis of fever. Considering the potential severity of undiagnosed infections, physicians may promptly escalate or broaden antimicrobial drugs, especially in patients recovering from life-threatening conditions despite the presence of alternative etiologies for fever. However, fever after ECMO decannulation in patients with critical cardiac or pulmonary conditions has not yet been systematically characterized. Practical information regarding the duration, peak, and impact of different fever etiologies on patient prognosis is lacking.

Therefore, we conducted an analysis to characterize fever after ECMO decannulation, identify the potential causative factors, and evaluate their associations with patient outcomes.

## 2. Materials and Methods

### 2.1. Study Population

This was a retrospective single-center study. We screened adult patients admitted to the ICU of Korea University Ansan Hospital between 1 March 2019 and 31 May 2024. Patients who underwent venoarterial (VA) or venovenous (VV) ECMO and were successfully weaned off ECMO during the study period were included. However, patients were excluded if their ECMO duration was < 48 h, if they were palliatively weaned off ECMO, or if they died within 48 h of ECMO discontinuation. Additionally, patients who received ECMO as a bridge to organ transplantation were excluded.

### 2.2. Data Collection

Demographic parameters, including age, sex, underlying diseases, and organ support schedule, were obtained from the electronic medical records. We recorded the peak body temperature daily for 16 days from the day before ECMO decannulation. Fever was defined as a body temperature > 37.8 °C, and temperatures < 36.0 °C were classified as hypothermia. “Decannulation fever” was defined as a fever that developed within 72 h of ECMO decannulation. Any fever that developed after three consecutive afebrile days was considered a new event unrelated to ECMO decannulation or a known febrile event. Infectious fever was defined as fever that developed in patients with an active infection, and non-infectious fever was defined as fever without any indication of active infection. The active infection was retrospectively assessed by focusing on the infection source considering culture results, imaging studies, and inflammatory markers comprehensively by two physicians. Positive results of blood culture, new organisms in sputum culture with increasing inflammatory markers and/or newly developed consolidation on chest radiography, and positive urine culture showing > 10^5^ colony-forming units with an increase in inflammatory markers were regarded as active infection conditions irrespective of fever or shock. When the cultured organisms were not new, serially increasing inflammatory markers with new opacities on chest radiography were defined as active infections. A positive result for Aspergillus Ag or CMV viremia was considered an active infection, considering the level, chest radiographic findings, and changing patterns of inflammatory markers. We used white blood cell count, neutrophil (%), C-reactive protein, and procalcitonin levels as inflammatory markers. We checked the presence of shock based on lactic acid level > 2 mmol/L and the requirement of a vasopressor for > 6 h and evaluated the association between shock and active infection considering the temporal correlation of events. The antibiotics administered during the study period were also recorded.

### 2.3. ECMO Management

ECMO was initiated in patients with severe circulatory or respiratory failure despite optimal medical management, as determined by a multidisciplinary team comprising an intensivist, a cardiothoracic surgeon, and the attending physician responsible for each patient. ECMO was performed by a cardiothoracic surgeon at the bedside of the patient, and the femoral–femoral approach was the main cannulation strategy for both VV and VA ECMO. The ECMO team provided ECMO-related patient care. ECMO decannulation was decided by the attending physician in consultation with the ECMO team based on the assessment that the affected organ had sufficiently recovered and that the patient could maintain a stable condition with minimal ECMO support. Once ECMO was decannulated, optimal medical care aimed at weaning patients from mechanical ventilation and other organ support was continued until the patient was discharged from the ICU.

### 2.4. Statistical Analysis

Continuous variables were expressed as mean ± standard deviation or median and interquartile range (IQR), depending on the data distribution. Categorical variables are expressed as frequencies and percentages. Comparisons between groups for continuous variables were conducted using the Wilcoxon rank-sum test, while categorical variables were compared using the χ^2^ test. Univariate logistic regression analysis was performed to identify the factors associated with in-hospital mortality. Multivariate logistic regression was then conducted on variables with a *p*-value < 0.1 from the univariate analysis, and on those deemed clinically significant. Confounding factors were excluded from the multivariate analysis, and significant factors were included irrespective of the *p*-value of the univariate analysis at the discretion of the authors. All statistical analyses were performed using the SPSS (version 20.0; IBM Corp., Armonk, NY, USA) and MedCalc software (version 22; MedCalc Software, Ostend, Belgium).

## 3. Results

### 3.1. Baseline Characteristics of the Patients

A total of 47 patients (30 men and 17 women, mean age 52 ± 17 years, all Asians) were successfully weaned from ECMO during the study period. Among them, 22 (46.8%) received VA ECMO and 25 (53.2%) received VV ECMO. The primary diagnosis in patients receiving VA ECMO was acute coronary syndrome (n = 11, 50.0%), whereas that in patients receiving VV ECMO was acute respiratory distress syndrome owing to pneumonia (n = 14, 56.0%).

The median ECMO duration was 9 (IQR: 6–19) days. Patients on VA ECMO had a shorter median duration of ECMO compared with that of patients on VV ECMO (8.5 [IQR: 4.75–11.75] vs. 11 [IQR: 7.5–28] days, *p* = 0.054). The length of hospital stay was significantly longer in patients on VV ECMO than in those on VA ECMO (58 [IQR: 31–127.5] vs. 25 (IQR: 16.75–78.25) days, *p* = 0.011). A total of 36 patients (76.6%) survived until discharge, 18 (81.8%) in the VA ECMO group and 18 (72%) in the VV ECMO group (*p* = 0.505) (Table 1).

### 3.2. Body Temperature Change After Decannulation

**Figure 1:**
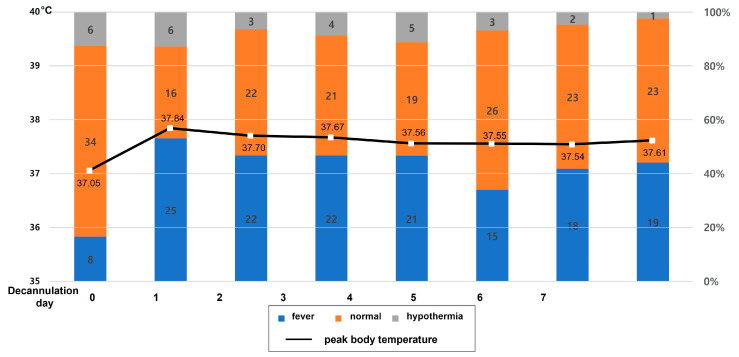
Number of patients with fever, normal temperature, and hypothermia, and mean peak body temperature of patients after extracorporeal membrane oxygenation decannulation.

**Figure 2:**
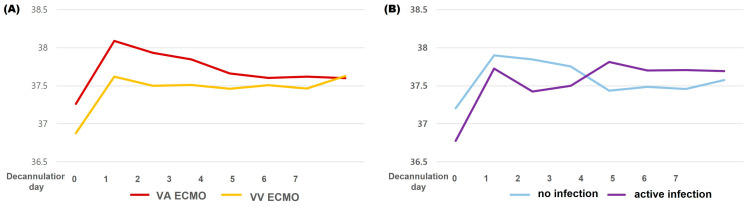
Changes in peak body temperature during the first 7 days from extracorporeal membrane oxygenation decannulation in patients with venoarterial and venovenous extracorporeal membrane oxygenation (**A**) and patients without active infection and with active infection (**B**).

Figure 1 illustrates the number of patients with fever, normothermia, and hypothermia recorded from days 0 to 7 after decannulation, along with the daily peak body temperature. During the 7 days, only five patients did not develop fever. The highest peak fever was observed on the first day of decannulation (37.8 [IQR: 37.3–38.1] °C) with the highest number of patients with fever (n = 25, 53.2%). Decannulation fever lasted until a median of decannulation day 5 (IQR: 2–10) and was more frequently observed in patients on VA ECMO than in those on VV ECMO (20 [90.9%] vs. 15 [60.0%], *p* = 0.020). Hypothermia developed in nine (14.9%) patients: three (13.6%) in the VA group and six (24%) in the VV group (*p* = 0.470). Peak body temperature was significantly higher in the VA ECMO group than in the VV ECMO group during the first 3 days after decannulation (Table 1, Figure 2A). No significant relation was observed between fever and hypothermia before or after decannulation (*p* = 0.412 and *p* = 0.322, respectively). The development of decannulation fever was not associated with the duration or setting of ECMO, and there were no significant differences in ECMO duration, flow rate, sweep gas rate, or pump speed between patients with and without decannulation fever (*p* = 0.961, *p* = 0.111, *p* = 0.166, and *p* = 0.602, respectively). The survival rate was higher in patients with decannulation fever than in those without decannulation fever (30/35 [85.7%] vs. 6/12 [50.0%], *p* = 0.020).

### 3.3. Infectious Condition of Patients and Fever

During the 7 days after decannulation, 16 patients were in an active infectious condition: four developed bloodstream infection, seven experienced the progression of previously diagnosed infectious disease, four developed new-onset pneumonia, and one developed a urinary tract infection; three patients developed septic shock among the six who developed shock after decannulation. No significant association was found between decannulation fever and infectious conditions (25/31 [80.6%] vs. 10/16 [62.5%], *p* = 0.289 [% of patients with decannulation fever among patients without and with active infection, respectively]) in these patients. Decannulation hypothermia was associated with infectious conditions (3/31 [9.7%] vs. 6/16 [37.5%]; *p* = 0.045). Although the peak body temperature was initially lower in patients with active infection during the first 3 days after decannulation, it tended to increase over time (Figure 2B). Fever that developed in patients with active infection lasted longer than that in those without active infection (4 days [IQR: 1–7] vs. 11 days [IQR: 2–17], *p* = 0.048), and fever that lasted > 7 days after decannulation was significantly associated with active infection (odds ratio [OR] 11.44, 95% confidence interval [CI] 2.749–47.615, *p* = 0.001).

Leukocytosis and elevated C-reactive protein (CRP) levels were common in patients receiving ECMO irrespective of the infection state in the peri-decannulation period. However, in patients without active infection, these markers tended to normalize, whereas in those with active infection, these increased further. Notably, CRP level on day 7 after decannulation was significantly higher in patients with an infectious condition (2.71 [1.44–7.42] vs. 8.61 [3.69–13.00], *p* = 0.006).

The presence of active infection was not associated with the type of ECMO used or duration of ECMO support (VA ECMO %, 16/31 [51.6%] vs. 6/16 [31.3%], *p* = 0.538; duration of ECMO, 9 [IQR: 6–19] vs. 10 [IQR: 4.25–18.25] days, *p* = 0.982, no active infection vs. active infection respectively). The ICU stay (19 [IQR: 13–46] vs. 27.5 [IQR: 12–49] days, *p* = 0.955) and hospital stay (32 [IQR: 21–91] vs. 67 [IQR: 32–96.75] days, *p* = 0.185) were longer in patients with active infections, but the difference was not statistically significant.

However, in-hospital mortality was significantly associated with the presence of an infectious condition in the first week after decannulation (4/31 [12.9%] vs. 7/16 [43.8%], *p* = 0.029).

Despite active infection in only 16 patients, 31 patients (23/31 [74.2%] without active infection and 8/16 [50%] with active infection, *p* = 0.117) underwent changes in their antibiotic regimen to cover potential pathogens within 3.5 (IQR: 2–5.75) days after decannulation, which did not significantly affect patient survival (Table 2).

### 3.4. Logistic Regression Analysis for In-Hospital Mortality

In the univariate analysis, decannulation fever was related to lower in-hospital mortality (OR 0.167, 95% CI 0.038–0.729, *p* = 0.017), whereas decannulation hypothermia was associated with higher in-hospital mortality (OR 6.667, 95% CI 1.376–32.290, *p* = 0.018). Decannulation hypothermia was also associated with active infection during this period. Active infection was associated with an increased in-hospital mortality (OR, 5.250; 95% CI, 1.242–22.194; *p* = 0.024). VV versus VA ECMO, duration of ECMO, presence of multidrug-resistant organisms, and antibiotic changes after decannulation were not significantly associated with in-hospital mortality. In multivariate analysis, decannulation was associated with lower mortality and active infection was related to higher in-hospital mortality (OR: 0.156, 95% CI: 0.025–0.997, *p* = 0.047; OR: 6.067, 95% CI: 1.128–32.644, *p* = 0.036) (Table 3).

## 4. Discussion

Fever commonly occurs after ECMO decannulation. However, decannulation fever was difficult to predict, and the etiology was diverse; it did not refer only to the infectious condition of the patients and was not related to mortality. Conversely, in patients with active infection that progressed during the post-decannulation period, the body temperature tended to gradually increase, and the fever lasted longer with higher CRP levels than in patients without active infection. Finally, active infection after decannulation was associated with higher mortality rates. Therefore, a focused evaluation of the signs of active infection might be helpful when considering the characteristics of infectious fever in these patients.

### 4.1. Fever After ECMO Decannulation

Fever is common in ICU and often signals an infectious disease [1,9,10]. However, various non-infectious causes can also contribute to fever in critically ill patients, such as acute coronary syndrome, transfusion reactions, drug fever, and adrenal insufficiency [2]. However, physicians generally treat fever in ICU patients with a strong suspicion of infection because infectious complications can be fatal in ICU patients. Furthermore, the presence of fever in patients who are weaned off ECMO often raises concerns because many are exposed to microbial organisms before decannulation [11,12]. In particular, patients on VV ECMO for acute respiratory distress syndrome are at a high risk of ventilator-associated pneumonia [13]. Similarly, patients on VA ECMO showed a high prevalence of pneumonia and bloodstream infections [14].

Nevertheless, non-infectious SIRS is also a predictable condition during and after ECMO. The ECMO circuit can trigger SIRS of various etiologies. The immune system is activated when blood interacts with the non-endothelialized surface of the ECMO circuit [5], leading to elevated levels of proinflammatory cytokines and activated complement system, which can result in leukocytosis [15,16,17]. Additional factors, such as thrombus formation in cannulated vessels or inflammation at the puncture site, may also contribute to fever [14].

Similar to patients who develop fever after the discontinuation of continuous renal replacement therapy, ECMO decannulation may lead to fever, reflecting a patient’s hypothermic state or delayed thermoregulatory adjustment [18]. Because the large volume of blood circulating through the circuit during extracorporeal circulation leads to heat loss [19,20], the human thermoregulatory system adapts to maintain a stable body temperature on ECMO. Therefore, after the cessation of extracorporeal circulation, the body may fail to maintain a normal temperature, resulting in fever. The use of a heat exchanger during ECMO may further complicate the interpretation of post-decannulation temperature changes. Research at the laboratory level is needed to confirm this pathophysiology.

VA and VV ECMO differ in their physiological and clinical characteristics. VA ECMO returns oxygenated blood directly to the arterial system, typically via the common iliac artery, bypassing pulmonary circulation. In contrast, VV ECMO returns blood to the right atrium, allowing it to pass through the pulmonary system before reaching the systemic circulation and peripheral organs [21]. This means that in VA ECMO, thrombi or proinflammatory cytokines generated by ECMO cannulas can be directly delivered to peripheral organs, triggering SIRS. Because ECMO requires large cannulas to remain in the vessels, the risk of thrombosis remains despite anticoagulation [22,23].

### 4.2. Clinical Characteristics of Patients with Decannulation Fever

The leukocytosis and elevated CRP levels observed before decannulation in this study reflect proinflammatory conditions induced by the ECMO circuit, which was similar to the results of other studies [8,24]. Decannulation fever developed in 74% of all patients in the present study, which was in the range of previously documented numbers from 44–84% [7,8,24,25]. The highest peak fever was observed on the first day of decannulation and gradually decreased [7,25]; non-infectious fever tended to develop within a day and lasted < 7 days (4 [IQR 1.5–7] days in this study) [7,24]. However, in contrast to those studies, our results showed a higher frequency of decannulation fever in patients receiving VA ECMO. This might be explained by the greater number of patients with active infection on VA ECMO, lower threshold for fever, different racial issues, or small sample size (Table 1, Figure 2A). Various factors can influence body temperature in these patients [8,18], but the development of non-infectious fever was unpredictable and was not associated with ECMO duration or other ECMO-related factors. Inflammatory markers such as leukocytosis and CRP were similarly elevated after decannulation, regardless of the infectious condition. Finally, it was not related to fatal outcomes; therefore, diagnosing infections that were related to higher mortality in these patients seems even more important.

### 4.3. Fever and Infection After ECMO Decannulation

In our evaluation, decannulation fever occurred in 74.5% of patients, yet only 34.0% had active infections during this period. This implies that decannulation fever is not primarily associated with infectious conditions after decannulation. Hypothermia within 3 days of decannulation was linked to active infection (*p* = 0.045) and higher mortality (Table 3), consistent with a previous report [7]. Instead, infectious fever showed a delayed peak that lasted for more than 11 (IQR: 2–17) days after decannulation. Fever detected > 7 days after decannulation was uncommon in patients without any evidence of active infection. CRP levels were also significantly higher in patients with active infection until 7 days after decannulation (Table 2). Importantly, active infections were associated with worse outcomes, emphasizing the need for physicians to carefully differentiate between infectious and non-infectious causes of fever in these patients.

In response to fever, most patients in this study were administered new antibiotics to cover all possible organisms after decannulation. However, these changes did not significantly affect the patient outcomes. This implies that fever should not be the only evidence of active infection, but other conditions should be considered when approaching the fever of patients after ECMO decannulation. Although infection might be the most fatal etiology of fever with an impact on patient prognosis, considering the proportion of patients with active infection, a thorough evaluation of the patient should be provided.

### 4.4. Limitation

This study has some limitations. First, this was a single-center retrospective evaluation, and the number of patients included was small. In particular, ECMO-related management could be different between centers so thatthe different conditions at each center in managing ECMO should be considered when interpreting decannulation fever. Therefore, our findings have limitations that restrict direct application to the clinical practice. Second, we did not routinely check the central body temperature; instead, the tympanic temperature was used because of the limitations of the retrospective evaluation. Third, our study did not suggest specific criteria for diagnosing non-infectious fever among patients with decannulation fever. However, we demonstrated the risk of using only vital signs or SIRS criteria to diagnose infectious conditions. For the differential diagnosis of infectious conditions, a thorough evaluation of the patient’s condition to determine the source of infection was emphasized, especially when the fever lasted > 7 days.

## 5. Conclusions

Fever was common after ECMO decannulation with various etiologies. Decannulation fever tended to be more frequent in patients receiving VA ECMO than in those receiving VV ECMO but was not related to prior ECMO settings or duration and did not directly indicate infectious conditions. Finally, the decannulation fever could not be predicted. In contrast, fever and increased CRP level lasting > 7 days after decannulation were related to active infection, which was significantly associated with in-hospital mortality. Although decannulation fever did not indicate active infection, a focused evaluation for the possible infection should be underscored, especially when the fever persists longer after decannulation.

## Figures and Tables

**Table 1:** Demographic data of patients successfully weaned off extracorporeal membrane oxygenation.

	Total, (N = 47)	VA (n = 22)	VV (n = 25)	*p*-Value
Age (years)	52 (17)	52 (20)	52 (13)	0.997
Male, n (%)	30 (63.8)	16 (72.7)	14 (56)	0.766
Immunocompromised host, n (%)	6 (12.7)	0 (0)	6 (24)	0.023
Cause of ECMO				
Acute coronary syndrome, n (%)	11 (23.4)	11 (50.0)	-	
Other heart failures (myocarditis, cardiomyopathy, and others), n (%)	7 (14.9)	7 (31.8)	-	
ARDS due to pneumonia, n (%)	14 (29.8)	-	14 (56.0)	
other ARDS, n (%)	7 (14.9)	-	7 (28.0)	
Other cause, n (%)	8 (17.0)	4 (18.2)	4 (16.0)	
Fever before ECMO decannulation, n (%)	9 (19.1)	6 (27.3)	3 (12.0)	0.270
Hypothermia before ECMO decannulation, n (%)	6 (12.8)	2 (9.1)	4 (16.0)	0.670
Decannulation fever, n (%)	35 (74.4)	20 (90.9)	15 (60.0)	0.020
Decannulation hypothermia, n (%)	9 (19.1)	3 (13.6)	6 (24.0)	0.470
Fever days during 1st week	3 (1–5)	4 (1.75–5.25)	2 (1–3)	0.027
Days of decannulation fever	5 (2–12)	6 (1.5–12.75)	4 (2–7)	0.458
Last fever day from decannulation	6 (1–11)	7 (3–12.25)	4 (0.25–7.75)	0.061
Mechanical ventilation, n (%)	47	22 (100.0)	25 (100.0)	1.00
CRRT, n (%)	16 (34%)	4 (18.2)	12 (48.0)	0.063
CRRT during ECMO weaning, n (%)	7 (14.9)	2 (9.1)	5 (20)	0.423
Blood flow rate of ECMO (L/minute)	1760 (1254–1767)	15,530 (1175–2030)	2000 (1595–2500)	0.007
Sweep gas flow rate (L/minute)	1 (0.5–2)	1.50 (0.70–2.25)	1.00 (0–2)	0.054
Location of cannulation (inflow/outflow)		Femoral vein/Femoral artery, 22 (100)	Femoral vein/femoral vein, 19 (76) Femoral vein/Jugular vein, 5 (20)	
Duration of ECMO, d	9 (6–19)	8.5 (4.75–11.75)	11 (7.5–28)	0.054
Length of ICU stay, d	21 (13–44.5)	16.5 (13–28)	30 (12.5–66)	0.100
Length of hospital stay, d	42 (23.5–89)	25 (16.75–78.25)	58 (31–127.5)	0.011
In-hospital mortality	11 (23.4)	4 (18.2)	7(28.0)	0.505

ECMO, extracorporeal membrane oxygenation; ARDS, acute respiratory membrane oxygenation; CRRT, continuous renal replacement therapy; ICU, intensive care unit.

**Table 2:** Comparison of extracorporeal membrane oxygenation settings, inflammatory markers, and prognosis of patients by the active infectious condition. *, *p* < 0.05 when compared with the laboratory data of previous evaluations in the same group.

	No Active Infection (n = 31)	Active Infection (n = 16)	*p*-Value
Age, years	53.6 ± 17.1	50.4 ± 15.8	0.539
sex (M/F)	23/8	7/9	0.057
Heart rate (/min)	93 ± 16	110 ± 18	0.002
Mean blood pressure (mmHg)	77 (70–92)	84 (73–99.5)	0.317
Use of vasopressors	13 (41.9)	8 (50)	0.758
Lactic acid on d 1 (mmol/L)	1.32 ± 0.98	1.47 ± 0.59	0.596
Shock, n (%)	2 (6.5)	4 (25)	0.320
Immunocompromised host, n (%)	1 (3.2)	5 (31.3)	0.013
Steroid use during the peri-decannulation period	10 (32.3)	7 (43.8)	0.528
ECMO (VA), n (%)	16 (51.6)	6 (37.5)	0.538
Fever before ECMO decannulation, n (%)	8 (25.8)	1 (6.25)	0.138
Hypothermia before ECMO decannulation, n (%)	3 (9.7)	6 (37.5)	0.045
Decannulation fever, n (%)	25 (78.1)	10 (62.5)	0.289
Hypothermia after decannulation, n (%)	6 (19.4)	3 (18.8)	1.00
Fever days during 1st week, days	3 (1–5)	3 (1–5)	0.973
Duration of fever, days	4 (1–7)	11 (2–17)	0.023
Fever lasted > 7 days after decannulation	5 (16.1)	11 (68.8)	0.001
WBC before decannulation (/μL)	10,630 (8090–14,190)	11,750 (7625–14,630)	0.966
WBC, decannulation day 1 (/μL)	10,960 (9090–13,790)	12,520 (8432–15,445) *	0.598
WBC, decannulation day 7 (/μL)	9520 (7880–12,975)	9815 (8295–13,785)	0.641
Neutrophil count before decannulation (/μL)	8663 (6697–12,562)	9682 (5595–12,029)	0.911
Neutrophil count, decannulation day 1 (/μL)	8614 (7283–11,209)	9817 (6792–13,444)	0.559
Neutrophil, decannulation day 7 (/μL)	6565 (5101–11,951)	7207 (5087–13,809)	0.795
CRP before decannulation (mg/dL)	8.23 (1.92–13.91)	9.43 (4.59–13.58)	0.566
CRP, decannulation day 1 (mg/dL)	9.12 (1.54–15.82)	11.23 (4.77–21.99)	0.178
CRP, decannulation day 7 (mg/dL)	2.71 (1.44–7.42) *	8.61 (3.69–13.00)	0.006
Culture (+) of MDR organisms during study period, n (%)	15 (48.4)	12 (75.0)	0.121
Coverage of MDR organism during study period, n (%)	21 (67.7)	11 (68.7)	1.000
Changed/Added antibiotics during study period, n (%)	23 (74.2)	8 (50.0)	0.117
Pump speed before weaning (/min)	1532 ± 426	1617 ± 618	0.527
Blood flow rate (L/min)	1848 ± 619	2104 ± 794	0.168
Sweep gas flow rate (L/min)	1.31 ± 1.01	1.26 ± 1.32	0.687
Duration of ECMO, days	9 (6–19)	10 (4.25–18.25)	0.982
ICU length of stay, days	19 (13–46)	27.5 (12–49)	0.955
Length of hospital stay, days	32 (21–91)	67.5 (32–96.75)	0.185
In-hospital mortality, n (%)	4 (12.9)	7 (43.8)	0.029

ECMO, extracorporeal membrane oxygenation; VA, venoarterial; WBC, white blood cell; CRP, C-reactive protein; ICU, intensive care unit; MDR, multi-drug resistant.

**Table 3:** Logistic regression analysis of parameters for patients’ in-hospital mortality after ECMO decannulation.

	Univariate Analysis			Multivariate Analysis		
Parameters	Odds Ratio	95% Confidence Interval	*p*-Value	Odds Ratio	95% Confidence Interval	*p*-Value
Age	1.004	0.963–1.047	0.836			
Sex	1.011	0.248–4.117	0.988			
VV ECMO	1.750	0.435–7.036	0.431	1.143	0.178–7.361	0.888
ECMO duration	0.985	0.946–1.027	0.483			
Decannulation fever	0.167	0.038–0.729	0.017	0.156	0.025–0.977	0.047
Decannulation hypothermia	6.667	1.376–32.290	0.018			
Fever duration	0.928	0.816–1.055	0.254			
Active Infection	5.250	1.242–22.194	0.024	6.067	1.128–32.644	0.036
CRP post-decannulation day 1	1.043	0.970–1.123	0.256			
CRP at post-decannulation day 7	1.138	1.012–1.279	0.031			
Antibiotics escalation	2.864	0.538–15.247	0.218			
MDR organism	0.857	0.220–3.337	0.824	0.312	0.047–2.049	0.225

ECMO, extracorporeal membrane oxygenation; VV, venovenous; CRP, C-reactive protein.

## Data Availability

The data presented in this study are available upon request from the corresponding author owing to the data transportation policy of the institution.
